# Biotransformation and Detoxification of Xylidine Orange Dye Using Immobilized Cells of Marine-Derived *Lysinibacillus sphaericus* D3

**DOI:** 10.3390/md15020030

**Published:** 2017-02-08

**Authors:** Prabha Devi, Solimabi Wahidullah, Farhan Sheikh, Rochelle Pereira, Niteen Narkhede, Divya Amonkar, Supriya Tilvi, Ram Murthy Meena

**Affiliations:** 1 Bioorganic Chemistry Lab., Chemical Oceanography Division, CSIR-National Institute of Oceanography, Dona Paula 403004, Goa, India; solima@nio.org (S.W.); sfarhan512@gmail.com(F.S.); rpereira@nio.org (R.P.); divya.amonkar@nio.org (D.A.); supriyatilvi@nio.org (S.T.); ram@nio.org (R.M.M.); 2 CSIR-Indian Institute of Integrative Medicine, Mumbai 400053, India; nanarkhede@iiim.ac.in

**Keywords:** sponge-associated bacteria, biotechnological application, analytical biotechnology, alginate-immobilized bacteria

## Abstract

*Lysinibacillus sphaericus* D3 cell-immobilized beads in natural gel sodium alginate decolorized the xylidine orange dye 1-(dimethylphenylazo)-2-naphthol-6-sulfonic acid sodium salt in the laboratory. Optimal conditions were selected for decolorization and the products formed were evaluated for toxicity by disc diffusion assay against common marine bacteria which revealed the non-toxic nature of the dye-degraded products. Decolorization of the brightly colored dye to colorless products was measured on an Ultra Violet-Vis spectrophotometer and its biodegradation products monitored on Thin Layer Chromatographic plate and High Performance Liquid Chromatography (HPLC). Finally, the metabolites formed in the decolorized medium were characterized by mass spectrometry. This analysis confirms the conversion of the parent molecule into lower molecular weight aromatic phenols and sulfonic acids as the final products of biotransformation. Based on the results, the probable degradation products of xylidine orange were naphthol, naphthylamine-6-sulfonic acid, 2-6-dihydroxynaphthalene, and *bis*-dinaphthylether. Thus, it may be concluded that the degradation pathway of the dye involved (a) reduction of its azo group by azoreductase enzyme (b) dimerization of the hydrazo compound followed by (c) degradation of monohydrazo as well as dimeric metabolites into low molecular weight aromatics. Finally, it may be worth exploring the possibility of commercially utilizing *L. sphaericus* D3 for industrial applications for treating large-scale dye waste water.

## 1. Introduction

With advances in analytical biotechnology, we are witnessing the birth of sophisticated analytical tools for the separation, characterization, and quantification of complex biological products. Dyes and pigments are extensively used as they play an essential role in the textile, leather, plastic and paper industry [1]. The color of the dye is imparted by the presence of chromophores like -N=N-, -C=O, -C=N-, -C=C-, NO_2_ or the quinoid group [2]. The three most commonly used dyes are azo, anthraquinone and phthalocyanine [1]. Azo dyes, in particular, contain an azo group (-N=N-) which usually links two aromatic systems or aromatic heterocycles or enolizable aliphatic groups [3]. As the demands for these products increases, dyeing wastewater increases several folds [4], and these dye industrial effluents finally pollute water bodies, causing unconditional pollution hazards to aquatic ecosystem. Improper disposal of the dyes leads to the reduction in sunlight penetration in water bodies, causing a decrease in photosynthetic activities and the increased heterotrophic activity lowers dissolved oxygen levels, adversely affecting aquatic life. It is estimated that approximately 10,000 different dyes and pigments are used industrially and over 0.7 million tons of synthetic dyes are produced annually worldwide, of which azo dyes make up to about 70%–80% by weight of all known dyestuff in the world [5]. These xenobiotic compounds are reported to be non-biodegradable, toxic and hazardous [6]. The main disadvantage associated with the treatment of wastewater by conventional methods is the formation of toxic compounds resulting from the cleavage of chromophoric groups [7]. Green technologies to deal with this problem include adsorption of dye stuff on bacterial and fungal biomass [8,9]. Although it was thought that azo dyes are not completely biodegradable, efforts are being made and reports are available describing how decolorization of reactive dyes is catalyzed by pure bacterial isolates. *Pseudomonas* sp. [10], *Klebsiella pneumonia* [11], *Exiguobacterium* sp. [12], *Alcaligenes faecalis*, *Rhodococcus erythropolis* [13] and *Rhizobium radiobacter* MTCC 8161 [14] are a few such examples. Biotransformation using microorganisms are gaining importance as it is cost effective and environmentally friendly [15]. The use of bacterial cells immobilized in a suitable gelling agent is a suitable alternative for bioremediation as it is more efficient when compared to free cells [16].

The objective of the present study is to isolate and identify the sponge-associated bacteria, *Lysinibacillus sphaericus* D3, most efficient in decolorizing and detoxifying xylidine orange dye under optimum conditions (pH, temperature, salinity, medium strength, stationary/agitated conditions, repeated use of beads and bead concentration) in media prepared with seawater as well as distilled water. Structural characterization of biotransformed metabolites by Liquid Chromatography Electrospray Ionization-Tandem Mass Spectrometry (LC ESI-MS/MS) forms an important part of this study.

## 2. Results and Discussion

### 2.1. Xylidine Orange Dye and Decolorizing Bacteria

**Figure 1:**
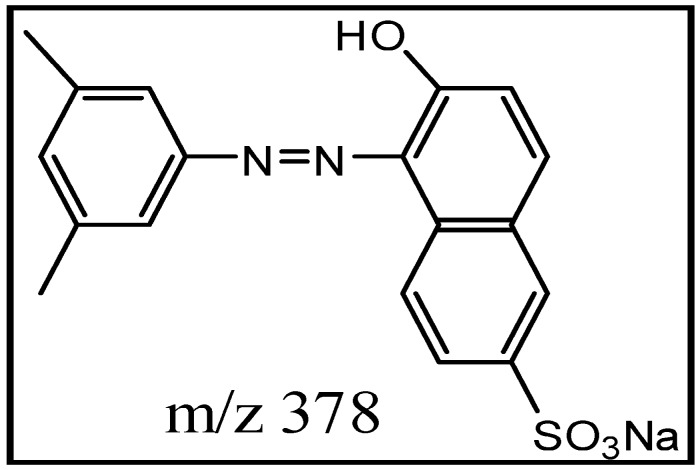
Structure of xylidine orange Dye.

**Scheme 1:**
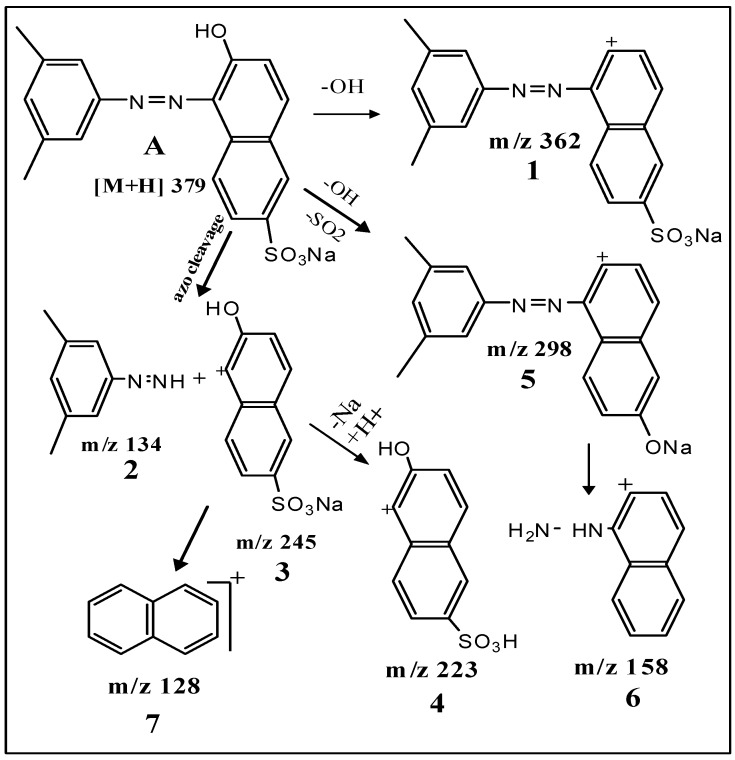
Fragmentation pathway observed in tandem mass spectrum of xylidine orange.

**Figure 2:**
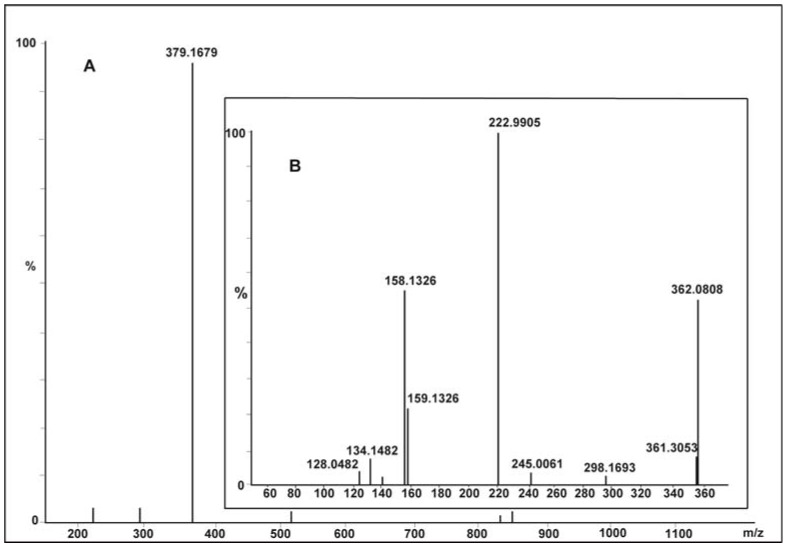
Positive Liquid Chromatography Electrospray Ionization Mass Spectrometry (LC ESI-MS) of xylidine orange dye (**A**) and tandem mass fragmentation (**B**) of [M + H]^+^ ion at *m*/*z* 379.

The xylidine orange dye currently used in the study is a 1-(dimethylphenylazo)-2-naphthol-6-sulfonic acid sodium salt (Figure 1), generously provided by a local dyeing industry from Gujarat. Since its structure was not known, we determined it by using data obtained from various analytical instruments, particularly LC ESI-MS/MS. The wide industrial application of this dye in textiles was mainly because it imparted brilliant orange/red color to fiber, possessing excellent fastness to washing. Its molecular mass was 378 Da (Figure 2A) and λ_max_ at 509 nm. Tandem mass (MS/MS) of [M + H]^+^ ions at *m*/*z* 379 displayed peaks at *m*/*z* 362, *m*/*z* 298, *m*/*z* 245, *m*/*z* 223, *m*/*z* 158, *m*/*z* 134 and *m*/*z* 128 (Figure 2B). Its structure was established on the basis of fragmentation observed on collision-induced dissociation (CID) of the parent molecule as shown in Scheme 1.

CID of the molecular ion at *m*/*z* 379 (Figure 2B; Scheme 1) produced product ion at *m*/*z* 362 (**1**) due to elimination of OH as water. Cleavage of azo bond yielded 3,5-dimethylphenyl hydrazine (**2**) (*m*/*z* 134) and sodium salt of 2-naphthol-6-sulfonic acid **3** (*m*/*z* 245). The base peak or the most intense signal at *m*/*z* 223 due the formation of 2-naphthol-6-sulfonic acid **4** results from elimination of sodium from **3**. Simultaneous elimination of hydroxyl and sulfur dioxide gave ion **5** at *m*/*z* 298. Further, cleavage of azo bond and elimination of NaOH from ion **5** produced naphthyl hydrazine **6** (*m*/*z* 158). Elimination of hydrazine from **6** or simultaneous elimination of –OH and desulfonation of **3**/**4** led to the formation of naphthalene ion **7** (*m*/*z* 128). The fragmentation observed was well in agreement with the structure of xylidine orange as 1-(dimethylphenylazo-2-naphthol-6-sulphonic acid sodium salt) **A**.

**Figure 3:**
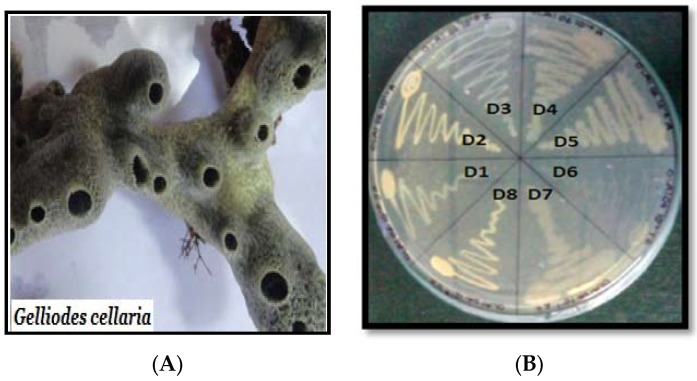
(**A**) *Gelliodes cellaria* (**B**) Associated bacteria.

Diverse groups of bacteria (*Pseudomonas luteola*, *Aeromonas hydrophila*, *Bacillus sublitis*, *Pseudomonas* spp. and *Proteus mirabilis*) from varying sources have been extensively studied to highlight their role in decolorization of different azo dyes [17] and in particular for the degradation of various sulfonated reactive azo dyes [18]. In the present study, we used marine-derived bacterium for decolorization and transformation causing degradation of xylidine orange dye. The said marine-derived bacterium was isolated from the surface of a marine sponge *Gelliodes cellaria* (Figure 3A). Sponges, in general, are sessile and filter-feeding metazoans, which host microorganisms comprising around 40%–60% of its biomass. Many of these sponge-associated bacteria, due to their unique characteristics, are preferred in biotechnological applications [19]. Out of the eight bacterial cultures isolated (Figure 3B), cultures D1, D3 and D7 showed potential decolorization activity. Culture D1 and D7 showed 8–15 mm clear zone around the disc while D3 showed a more than 16-mm clear zone. Since culture D3 was most efficient, it was selected for further investigation. Several studies of this kind were carried out using free bacteria in liquid medium. However, our results demonstrated that inoculation of the immobilized bacteria by gelling as beads using sodium alginate is a promising option for xylidine dye degradation studies. Lyophilized beads preserved for 1–3 months were also capable of decolorizing the said dye (8–10 h) as in the case of unlyophilized beads (8–10 h).

### 2.2. Identification of Dye Degrading Strain D3 by Polymerase Chain Reaction (PCR) Analysis

**Figure 4:**
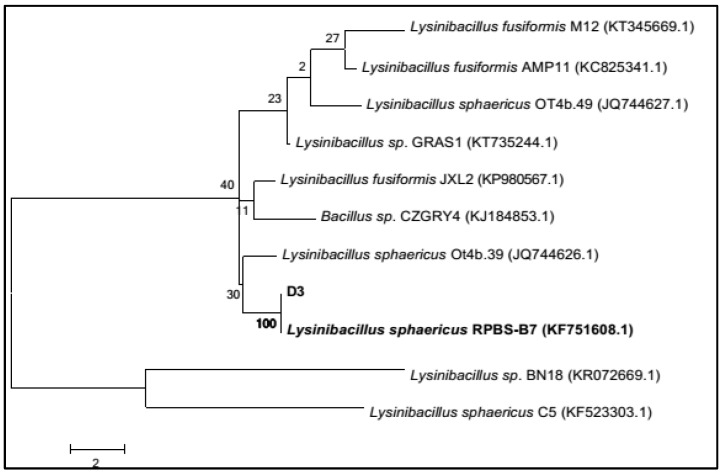
Phylogenetic tree based on 16S ribosomal RNA (16S rRNA) gene sequence showing the phylogenetic relationship between D3 and related strain for identification.

Culture D3 was identified to be *L. sphaericus* on the basis of nucleotide homology and phylogenetic analysis, showing 99% similarity to *L. sphaericus* RPBS-B7 (Figure 4). This further confirms the remarkable ability of marine sponges in harboring diverse microbes possessing wide applications [18].

### 2.3. Physico-Chemical Parameters for Xylidine Orange Decolorization

The optimization of physico-chemical parameters, as listed in Table 1, for enhanced decolorization of xylidine orange by *L. sphaericus* D3 was carried out one parameter at a time.

Full strength (13 g/L) nutrient broth medium prepared in diluted seawater (16 g/L), at pH 7.2, under shaker conditions (120 rpm) was most appropriate to obtain the degraded product. There was no difference in the decolorization rate at full and half strength (6.5 g/L) of nutrient broth (NB) medium. However, at quarter (3.25 g/L) and in 0% strength of media, decolorization was extremely slow and did not go to completion, indicating the importance of nutrients in the medium. 

**Figure 5:**
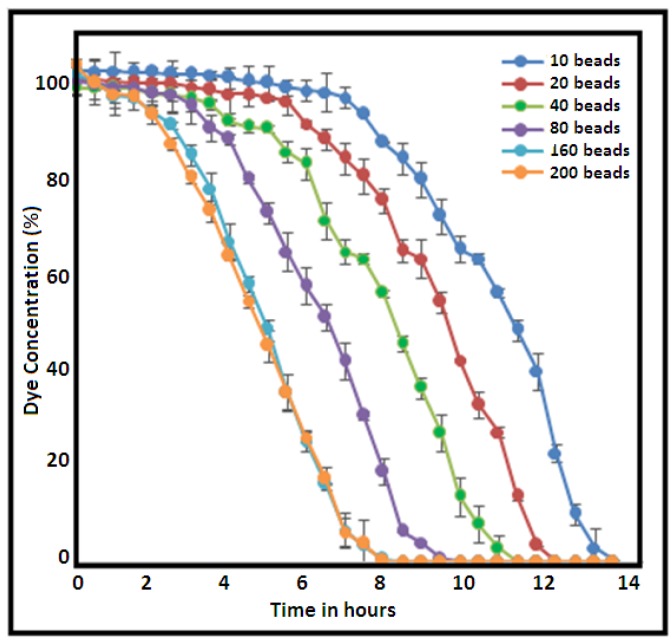
Degradation of dye in 500 mL medium with variation in *L. sphaericus* D3-immobilized bead numbers.

The medium for the decolorization experiment also worked well when the medium was prepared in distilled water. Hence, it was seen that *L. sphaericus* D3 was almost unaffected by saline conditions in the medium. A similar report on salinity tolerance was also made for *L. sphaericus* isolated from the marine sponge *Tedania anhelans* of the Tamil Nadu coast [20]. Furthermore, Rajeswari and co-workers (2014) [21] labeled *L. sphaericus* to be a very hardy strain capable of surviving under extremely harsh conditions. This culture is also known to be a producer of azoreductase [22] which can transform azo compounds to corresponding amines, hence making this bacterial isolate an ideal candidate for bioremediation both in fresh and saline waste waters containing nitro-aromatic and azo compounds. Additionally, it was also noted that increase in the bead number (10, 20, 40, 80, 160 and 200) did enhance activity when the volume of the medium used was kept constant (Figure 5). Beyond 160, no further decrease in time was noticed for decolorization of the dye.

During the experiments, the cells were immobilized in sodium alginate beads since immobilized cells had several advantages over suspended cells [16]. Alginate is a most widely used natural gel for cell entrapment based on its low cost, non-toxicity to the system, ability to retain high biomass content, good substrate diffusion and higher operational stability [23]. Each bead prepared was approximately of 4-mm diameter and contained approximately 2 × 10^6^ colony forming unit (CFU) of *L. sphaericus* D3. Previous study by Rajeswari et al. (2014) reports the degradation of textile dyes by *L. sphaericus* strain RSV-1 free cells within 24–30 h [21] for a dye concentration of up to 500 mg/L. However, the present investigation using immobilized cells of *L. sphaericus* D3 decolorized the same concentration of the dye within 8 h (Figure 5). This technique helped to arrest growth, keeping cells alive at a particular phase of growth for its application.

Furthermore, it was observed that the bacterial cells immobilized in sodium alginate remained active with the same efficiency after repeated use. We tried using the same beads successively three times. During the fourth use, the beads began to disintegrate, releasing cells into the medium. However, better efficiency of immobilized cells on repeated use was not observed as reported earlier by Djefal-Kerrar and group [24] who stated improved degradation efficiency of the culture during polyaromatic hydrocarbon (PAH) degradation studies, due to prolonged acclimatization of the cells towards the contaminants. It was also very interesting to note that alginate-encapsulated bacterial cells dried by lyophilization could be preserved by refrigeration at 4 °C for further use. These beads can be activated by immersing them in NB medium for 2 h prior to the start of the experiment. Such immobilized and stored cells can regain their viability upto several months (1–3 months) which can be an important parameter in current bioremediation technology.

### 2.4. Toxicological Evaluation of the Dye before and after Treatment

It was important to understand the toxic nature of the original native dye as well as its decolorized and degraded products. Few instances have reported the degraded products to be more toxic than the parent dye [25]. Hence, an attempt was made to determine the toxicity of the dye before and after its degradation based on simple disc diffusion assay against 12 common marine bacteria (Table 2). The non-toxic effect of the degraded product was evident from the absence of a zone of growth inhibition (halo) around the disc loaded with the degraded product (25 µg/disc) when compared with the disc loaded with control dye (25 µg/disc). From the 12 marine bacterial cultures used, *Bacillus aerophilus*, *Bacillus altitudinis* and *Bacillus pocheonensis* showed 2-mm, 3-mm and 1-mm halos respectively, formed around the xylidine orange dye. None of the other marine bacterial growth was affected by the native dye. On the other hand, none of the bacteria showed any halo around the discs when loaded with the degraded product (25 µg/disc). The experiment was performed in duplicates. This confirmed an environmentally safe method of azo dye degradation.

### 2.5. Spectral Analysis and Identification of Biotransformed Products

In order to examine post-treatment fate of the dye, the study of degradation product is extremely necessary. The detailed characterization of the intermediate and metabolite produced by biodegradation must be carried out to ensure the absence of toxic intermediates such as aromatic amines which are known to be carcinogenic [26].

On complete decolorization, various analytical tools were used to confirm degradation of the dye. It was clearly evident from the HPLC chromatogram (Figure S1) of the dye before (A) and after (B) bacterial treatment that the major peak with retention time 2.56 min of the dye was totally absent in the treated product. Similarly, absorption peaks evident at 206, 262, 315, 329 and 509 nm in the Ultra Violet-Vis absorbance spectrum (Figure S2) of the original brightly colored dye (A) are considerably reduced and practically absent in the visible range (B treated). For all decolorization experiments, optical density (OD) was preferably measured at 509 nm. Decolorization indicated cleavage of azo bond (-N=N-) resulting in breaking of conjugation effect. Microorganisms have the ability to produce enzymes capable of decolorizing and mineralizing azo compounds under suitable environmental conditions [19,20,21].

**Scheme 2:**
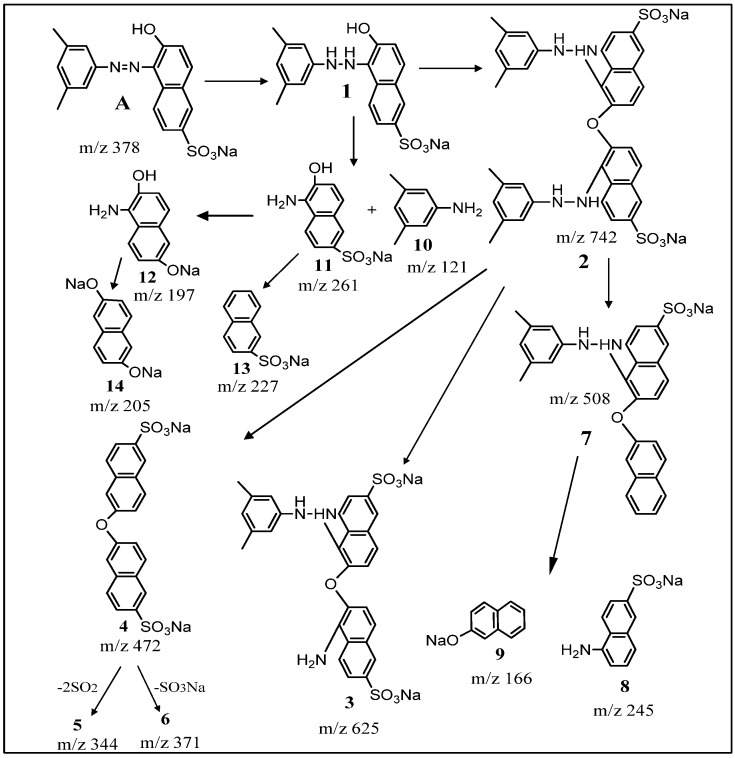
Proposed fragmentation pathway of degradation of xylidine orange dye by *L. sphaericus* D3 as analyzed by Liquid Chromatography Electrospray Ionization-Tandem Mass Spectrometry (LC-ESI-MS/MS).

LC ESI-MS profile of the decolorized products of xylidine orange dye (Figure S3) showed several protonated molecular ions, which on CID helped in identification of products formed after treatment with *L. sphaericus* D3 (Table 3). Accordingly, the biotransformed products formed and the schematic pathway of their formation is proposed in Scheme 2.

Two different pathways seem to be involved in the decolorization of xylidine orange into colorless non-toxic products (Scheme 2). Initially, dye **A**, (M + H)^+^ *m*/*z* 379 undergoes reduction of the azo group by azoreductase enzyme, resulting in the formation of hydrazo derivative 1-(dimethylphenylhydrazine)-2-naphthol-6-sulfonic acid-sodium salt **1**. Condensation of two molecules of **1** produces dimer **2** (*m*/*z* 742). The presence of its sodium adduct was also evident at *m*/*z* 764 (Figure S3). Formation of **1** and **2** is followed by their degradation into low molecular weight aromatics. Thus, the dimerichydrazo compound **2** undergoes biotransformation to yield products (**3**–**9**). Briefly, cleavage of one hydrazo bond by azoreductase enzyme resulted in the formation of **3** (*m*/*z* 625) with elimination of 3,5 dimethylaniline. Reductive elimination of two dimethylphenylhydrazine molecules from the dimer **2** produced 2,2′-dinaphthyl ether-6,6′-disulfonic acid disodium salt **4** with molecular ion peak at *m*/*z* 472. Compound **4** undergoes desulfonation (-SO_3_Na and -2SO_2_), resulting in the formation of products **5** and **6**, corresponding to *m*/*z* 344 and *m*/*z* 371, respectively. The dimer **2** also undergoes desulfonation (-SO_3_Na) and simultaneous reductive elimination of one molecule of dimethylphenylhydrazine to produce **7** corresponding to the mass of molecular ion peak at *m*/*z* 508. Metabolite **7** undergoes oxidation-reductive cleavage of ether and hydrazo bond to yield 1-naphthylamine-6-sulfonic acid sodium salt **8** with *m*/*z* 245 and 2-naphthol sodium salt **9** with *m*/*z* 166 (Table 3). Similarly, reductive cleavage of **1** produced dimethylaniline with molecular mass of *m*/*z* 121 (**10**) and 1-amino-2-naphthol-6-sulfonic acid sodium salt **11** with *m*/*z* 261, which undergoes desulfurization to yield product **12**. Deamination and dehydration of **11** resulted in the formation of sodium salt of naphthalene 2-sulfonate **13** with *m*/*z* 227. Deamination of **12** resulted in disodium salt of 2,6-hydroxy naphthalene **14** molecular ion with *m*/*z* 205.

**Scheme 3:**
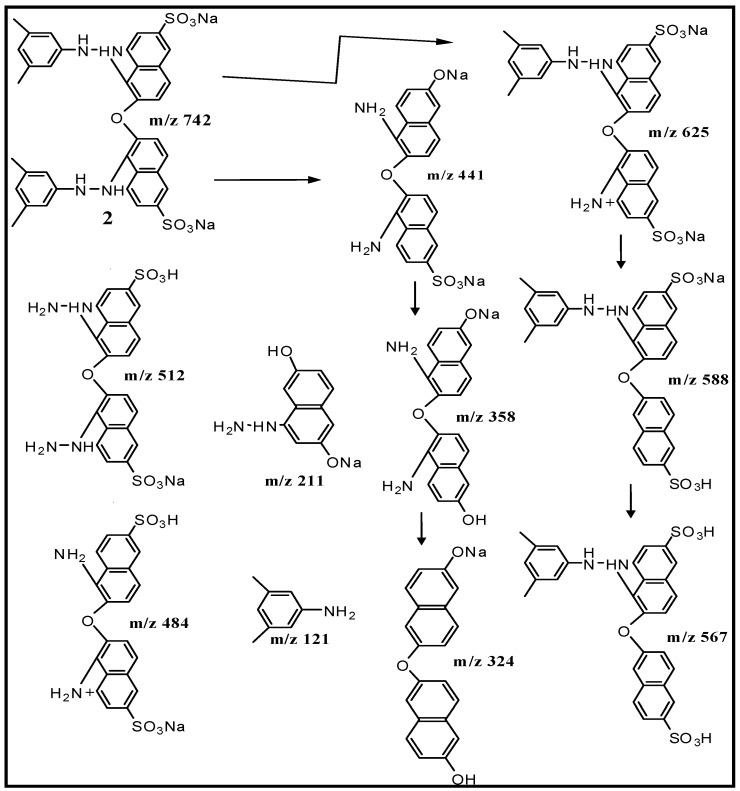
Identification of dimer (**2**) as one of the biotransformation products of xylidine orange from tandem mass (MS/MS) fragmentation of *m*/*z* 742 data.

The structure of dimer **2**, one of the products of biotransformation was confirmed by CID of its molecular ion at *m*/*z* 742 (Figure S4). Formation of daughter ions at *m*/*z* 625, *m*/*z* 588, *m*/*z* 567, *m*/*z* 512, *m*/*z* 484, *m*/*z* 441, *m*/*z* 358, *m*/*z* 324, *m*/*z* 211, *m*/*z* 169 and *m*/*z* 121 could be easily explained on the basis of fragmentation pattern observed as illustrated in Scheme 3. Hence, decolorization mainly takes place due to the production of azoreductases by *L. sphaericus* D3 which can specifically catalyze the reduction of azo (-N=N-) bond in azo dyes to produce colorless amines.

## 3. Materials and Methods

### 3.1. Dye and Reagents Used in the Study

Xylidine orange, a mono azo dye with molecular formula of C_18_H_15_N_2_O_4_SNa, and chemically 1-(dimethylphenylazo)-2-naphthol-6-sulfonic acid sodium salt (Figure 1) was selected as a model dye. It is an extensively used dye and was gifted by a textile dyeing industry from Gujarat. All other reagents/solvents used were of analytical grade and procured from HiMedia Pvt. Ltd., Mumbai, India.

### 3.2. Analytical Techniques/Instrumentation

Thin-layer chromatography (TLC) of the control dye and decolorized/degraded products was performed on silica gel coated aluminum sheets (60 F_254_ Merck, Darmstadt, Germany). Spots were developed using methanol:chloroform (5:95%) as solvent system and visualized as fluorescent spots under UV light at 366 nm. Gradual change in the color of the dye with time was monitored on UV-Vis Spectrophotometer (UV-240IPC, Shimadzu Instrument, Kyoto, Japan) at 509 nm. The percentage of dye in the medium was calculated from the calibration factor by calibrating standard dye of known concentration. The HPLC profile of the original dye and the decolorized product was performed on HPLC (Waters 2535, Milford, MA, USA) using C18 Xbridge 5 µm (4.6 × 250 mm) column at 25 °C. The gradient system comprised of water: acetonitrile (50:50 at time 0 to 0:100 after 10 min) with the flow rate of 1 mL/min monitored at wavelength 254 nm using a UV detector. Probable transformation products formed were analyzed by LC ESI-MS/MS on an instrument from WATERS (Model-ACQUITY Xevo-TQD, Milford, MA, USA) in positive ion mode [UPLC column: C_18_ (1.7 m, 2.1 × 50 mm); MS/MS parameters: source temperature 150 °C, desolvation temperature 450 °C, source voltage for capillary 3 kV, cone 30 V, source gas flow: desolvation 1000 L/Hr, cone 20 L/Hr, collision energy 20 V]. The lyophilized samples dissolved with methanol:water (1:1) were directly infused into the system.

### 3.3. Isolation of Sponge-Associated Bacteria Capable of Dye Decolorization

During the course of our on-going research program on screening sponge-associated bacteria for biotechnological application, the sponge sample, *Gelliode scellaria* (Figure 3A) was collected from the intertidal region of Mandapam on the South-Indian coast (9.2800° N, 79.1200° E) in July 2014. Standard procedure as reported earlier was used for the isolation of sponge-associated bacteria [27]. Pure cultures (Figure 3B) were stored on slants at 4 °C until use.

The culturable sponge-associated bacteria were grown (drop culture) on nutrient agar (NA, HiMedia, Mumbai, India) comprising of 5 g/L of peptic digest of animal tissue, 5 g/L of NaCl, 1.5 g/L of beef extract, 1.5 g/L of yeast extract and 20 g/L of agar prepared in 16 g/L seawater supplemented with 30% xylidine dye). The pH of the medium was 7.2 and temperature was maintained at 28 °C. Agar plates were incubated for 24 h and observed for the formation of clear decolorized zone around the drop culture. Decolorization was ranked on the basis of visual identification of the zone in mm, (0 mm) no decolorization, (2–7 mm) slight decolorization, (8–15 mm) moderate decolorization and (>16 mm) intense decolorization. For all decolorization experiments the bacterial culture (D3) was grown in liquid nutrient broth medium (NB HiMedia, ingredients same as above for NA but without agar) at 28 °C on a shaker at 120 rpm.

### 3.4. Immobilization of Marine-Derived Bacterium

On using free and immobilized bacterial cells in decolorizing the dye, the latter yielded better results [23]. Hence, introduction of microbial inoculants into experimental flasks in the immobilized form as alginate beads was preferred. In view of this, immobilization of bacterial culture (D3) by entrapment in 3% sodium alginate solution was carried out according to a modified procedure of Bashan [28]. Briefly, a loopful of the most efficient culture (D3) in decolorizing azo dye was inoculated into 250 mL Erlenmeyer flask containing 100 mL of NB broth medium (as described above). Flasks were incubated on an orbital shaker (Labnet, ORBIT LS, Edison, NJ, USA) at 80 rpm and fermented at 32 °C for 48 h (until the early stationary phase). At the end of the incubation period, culture medium was centrifuged at 6000 rpm for 15 min to collect the cell pellet. Entrapment of bacterial cells within beads was carried out under sterile conditions in the laminar air flow cabinet. Suspension of cell pellets in 10 mL of sterile filtered seawater (SFSW, 16–20 g/L) was added to 40 mL of sodium alginate solution prepared in distilled water. The final suspension contained 3% alginate by weight and 4 × 10^8^ cfu/mL of *L. sphaericus*. The mixture was well stirred to avoid clump formation and dripped into a flask containing 4% calcium chloride solution for gelling. The flask into which the beads were dropped was gently rotated by placing it on a shaker. The beads (~4 mm diameter) were freed of CaCl_2_ by washing them repeatedly with sterile distilled water and stored in sealed sterile plastic containers at 4 °C until use. 

### 3.5. 16S Ribosomal Ribonucleic Acid (rRNA) Gene Sequencing of the Dye Degrading Strain D3

DNA extraction, PCR amplification of 16S rRNA gene of D3 culture was carried out as described earlier [29]. Briefly PCR amplification was performed in a total volume of 25 µL using qTaq PCR master mix (qARTA Bio. Inc., Carson, CA, USA), using 1 µL of the universal primer and 1 µL of template DNA, a forward primer 27F (5′AGAGTTTGATCCTGGCTCAG3′) and a reverse primer 1492R (5′ TACGGYTACCTTGTTACGACTT3′). PCR amplification product was purified using the AxyPrep^TM^ (Axygen, New York, NY, USA) PCR cleanup kit according to manufacturer’s protocol. Quantification of nucleic acid was done using Thermo Scientific Nanodrop 2000R, USA. Sequencing of the PCR product was done using 3130XL Genetic analyzer (Applied Biosystems^TM^, Carlsbad, CA, USA). The percentage similarity was obtained by comparing with existing 16S rRNA gene sequence in the National Centre for Biotechnology Information (NCBI) database using Basic Logic Alignment Search Tool (BLAST) available at http://www.ncbi.nlm.nih.gov/blast. A total of 1403 bases and the nearest homolog obtained were aligned using Clustal W. The evolutionary history was inferred by using the maximum likelihood method based on the Jukes–Cantor model [30]. Evolutionary analyses were conducted using software Molecular Evolutionary Genetics Analysis (MEGA7) [31]. The bootstrap analysis was used based on 500 re-samplings. The nucleotide sequence was deposited in the GenBank database (sequence no. KU375125) and the culture was deposited at Microbial Culture Collection (MCC) of the National Centre for Cell Science (NCCS), bearing accession no MCC2951.

### 3.6. Optimization of Culture Media for Dye Decolourization 

A series of experiments were conducted to optimize the culture condition of D3 to achieve highest decolorization efficiency of the dye (Table 1). The following parameters were studied: effect of pH (5 to 11), salinity (0 to 25 g/L), static and shaker (120 rpm) conditions, solid and liquid medium; different concentrations of nutrient broth medium i.e., full strength (as described above) half strength, quarter strength and zero strength media were used as culture medium and amended with xylidine orange dye to make a final dye concentration of 300 mg/L using NB Media, diluted in seawater of 16 g/L salinity/distilled water [10]. Experiments were also run to record decolorization of dye in different flasks containing varying numbers of immobilized beads ranging from 10, 20, 40, 80, 160 and 200 beads per 500 mL (Figure 5). Another set of experiment was run to decolorize dye using reused beads as well as using lyophilized and stored beads [23]. Since freeze-dried bacteria (lyophilization) is a well-established method for long term storage of cells, a similar method of lyophilizing beads was performed using Heto DRYWINNER from Thermo Scientific, provided by a vacuum pump and a condenser. Low temperature (−50 °C) and vacuum (60 µbar) for 8 h aided in removing excess water. Such lyophilized beads were sealed in sterile containers and stored at −4 °C for 1–3 months until use. In all the experiments decolorization of dye was monitored spectrophotometrically by reading OD at 509 nm. Control flasks contained beads without bacterial culture. All experiments were carried out in triplicate under identical conditions. The concentration of dye in the medium was determined by initially calibrating the method using different standard concentration of dye. The absorption (OD-reading) obtained as a function of concentration helped to determine the unknown concentration using calibration factor.

### 3.7. Extraction of Dye-Degraded Metabolites

Upon optimizing the most suitable condition for dye decolorization using cells of *L. sphaericus* D3 (Table 1), the experiment was repeated on a larger scale (2L) in order to obtain sufficient material for analyzing the degraded products. For this purpose, experimental flasks containing dye and beads with culture while control flasks contained dye and beads without culture were harvested until complete decolorization of medium (dye, 300 mg/L) was observed in experimental flasks. The cell-free fermentation medium was partitioned with *n*-butanol. Extraction was repeated thrice and the combined extract concentrated under vacuum to obtain the crude decolorized biotransformed product. The metabolites were subjected to analysis by UV-Vis, HPLC and LC ESI-MS/MS.

### 3.8. Toxicity Evaluation of the Dye and the Biodegraded Product

Ecotoxicological tests should be conducted during the biodegradation process to ensure that the products of degradation are less toxic than the original pollutants. Considering that decolorization is not always an indication of successful detoxification, toxicity tests with the dye before and after decolorization were performed against some common marine bacteria using standard disc diffusion assay (Table 2). Briefly, the extract (25 µg/disc) from the control flask and transformed product (25 µg/disc) were dissolved in methanol and loaded onto sterile paper discs (6 mm diameter). The discs were allowed to dry in the laminar air flow and mounted onto nutrient broth agar plates on which a lawn of selected marine bacterium was uniformly spread plated. The plates were incubated for 24 h at 28 °C to visualize the zone of growth inhibition formed around the discs.

## 4. Conclusions

Microbial transformation causing decolorization of azo dyes is an efficient alternative tool for environmental management. The information obtained from this study may be useful for further development of bacterium *L. sphaericus* D3 as a biocatalyst for the effective decolorization, degradation and detoxification of xylidine orange dye before its release into the environment. This is the first report on the efficient biotransformation of xylidine orange dye using *L. sphaericus*. The use of modern instrumentation technique using tandem mass data gave fragment ions which were diagnostic in determining the products formed (Table 3). The non-toxic effect of the product was provenon marine bacteria collected from coastal waters. The method of using *L. sphaericus* D3 cells immobilized in natural gel alginate beads was stable for longer periods, and with this method large quantities of alginate beads can be prepared and stored in sterile containers for use as and when required (with prior revival of the beads in NB medium 2 h before use). They can also be used repeatedly (at least three times) without change in their efficiency, making this study an important contribution to safe wastewater management in industrial applications. Decolorization of xylidine orange dye by *L. sphaericus* D3 results from the reduction of azo group of the dye by azoreductase enzyme, condensation of the hydrazo compound to produce dimer, followed by degradation of both mono as well as dimerichydrazo derivatives.

## Figures and Tables

**Table 1:** Conditions in which the cells of *Lysinibacillus sphaericus* decolorize xylidine orange dye and the optimum conditions in which the present study was performed.

Parameters	Condition Causing Decolorization	Optimum Conditions
Free/encapsulated cells	Free as well as encapsulated	Encapsulated in alginate beads
Medium: liquid/solid	Both types	Liquid Nutrient Agar (NA) medium
pH	5–11	7.2
Temp	26–34 °C	28 °C
Saline/fresh water	0–25 g/L	16 g/L
Medium strength	Half/full strength	Full strength
Shaking/stationary	Both conditions	Shaker at 120 rpm
Reuse of beads	Upto threeuses	First use
Number of beads	Optional(3–35 beads/100 mL)	27 beads/100 mL
Use of unlyophilized/lyophilized beads	Both produced results (8–10 h) Lyophilized beads stored for 1–3 months	Cells aged one week

**Table 2:** Toxicity screening of xylidine dye (25 µg/disc) against randomly selected marine bacteria.

Marine Bacterium (Gram Characteristics)	Dye (Untreated)	Treated Dye
*Bacillus* sp. LTW29 [G+]	-	-
*Bacillus aerophilus* strain IHB B15224 [G+]	+ (2 mm)	-
*Bacillus altitudinis* strain BCX-57 [G+]	+ (3 mm)	-
*Bacillus* sp. L219 [G+]	-	-
*Bacillus pocheonensis* BCX-16 [G+]	+ (1 mm)	-
*Bacillus flexus* strain TAX4 [G+]	-	-
*Oceanobacillus oncorhynchi* strain AM-71 [G+]	-	-
*Novosphingobium barchamii* strain LL02 [G-]	-	-
*Vibrio natriegens* strain F7S194 [G-]	-	-
*Halomonas* sp. WHB35 [G-]	-	-
*Pseudovibrio* sp. 010K 105-5-5 [G-]	-	-
*Paracoccusyeei* strain G1212 [G-]	-	-

- no halo around the disc; + halo formed around the disc., G+ Gram positive, G- Gram negative.

**Table 3:** Fragmentation of degradation products of xylidine orange as observed by LC ESI-MS/MS.

Product	Mass/Charge (*m*/*z*)	Collision Energy (V)	*m*/*z* (% Relative Intensity)
Dye alone (**A**)	[M + H]^+^ 379	3.34 × 10^7^	379, 362, 298, 223, 158, 156, 128
Dimer (**2**)	[M + H]^+^ 743	4.69 × 10^5^	742, 724, 625, 588, 567, 512, 484, 441 (100%), 358, 324, 211, 169, 120
(**4**)	[M + H]^+^ 472	1.29 × 10^7^	472, 227
(**5**)	[M + H]^+^ 344	3.60 × 10^7^	344, 211, 212
(**7**)	[M + H]^+^ 508	2.38 × 10^7^	508, 439, 245, 246
(**8**)	[M + H]^+^ 245	1.10 × 10^7^	245, 217, 172, 154, 120 (100%), 98, 86, 70
(**9**)	[M + H]^+^ 166	1.25 × 10^8^	166, 167
(**13**)	[M + H]^+^ 227	1.56 × 10^7^	227, 197
(**14**)	[M + H]^+^ 205	1.69 × 10^7^	205, 206

## Supplementary Materials

The following are available online at www.mdpi.com/1660-3397/15/2/30/s1, Figure S1: HPLC chromatogram of dye before (A) and after (B) treatment with *Lysinibacillus sphaericus*, Figure S2: UV-Vis absorption spectra of the dye before (control) and after treatment (treated) with *Lysinibacillus sphaericus* D3, Figure S3: LC ESI MS of the degraded products showing different protonated molecular ions, Figure S4: Tandem mass spectrum(MS/MS) of the molecular ion at *m*/*z* 742, one of the products of biotransformation of the dye by *L. sphaericus*.
