# Supplementary material for: Biotransformation and Detoxification of Xylidine Orange Dye Using Immobilized Cells of Marine-Derived Lysinibacillus sphaericus D3
Source: Mar Drugs. 2017 Feb 8;15(2):30. doi: 10.3390/md15020030 (PMC5334610; doi:10.3390/md15020030)
Supplement: Supplementary file 1 [file marinedrugs-15-00030-s001.docx]

Supplementary Materials: Biotransformation and Detoxification of Xylidine Orange Dye Using Immobilized Cells of Marine Derived *Lysinibacillussphaericus* D3

Prabha Devi, Solimabi Wahidullah, Farhan Sheikh, Rochelle Pereira, Niteen Narkhede,
Divya Amonkar, Supriya Tilvi and Ram Murthy Meena


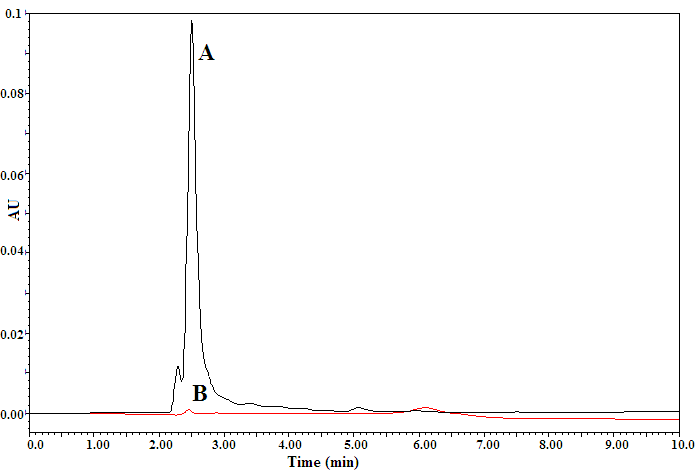


**Figure S1.** HPLC chromatogram of dye before (**A**) and after (**B**) treatment with *Lysinibacillus sphaericus* D3.


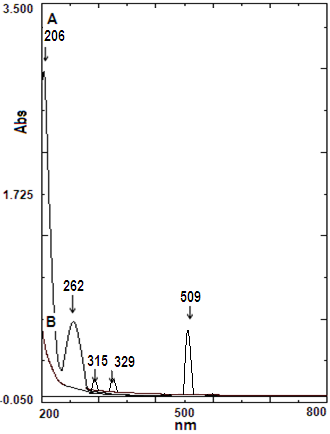


**Figure S2.** UV-Vis absorption spectra of the dye before (Control) and after treatment (Treated) with *Lysinibacillus sphaericus* D3.

**Figure S3.** LC ESI MS of the degraded products showing different protonated molecular ions.

**Figure S4.** Tandem mass spectrum (MS/MS) of the molecular ion at *m*/*z* 742, one of the products of biotransformation of the dye by *L. sphaericus*.
